# Modeling of senescence-related chemoresistance in ovarian cancer using data analysis and patient-derived organoids

**DOI:** 10.3389/fonc.2023.1291559

**Published:** 2024-02-02

**Authors:** Xintong Cai, Yanhong Li, Jianfeng Zheng, Li Liu, Zicong Jiao, Jie Lin, Shan Jiang, Xuefen Lin, Yang Sun

**Affiliations:** ^1^ Department of Gynecology, Clinical Oncology School of Fujian Medical University, Fujian Cancer Hospital, Fuzhou, Fujian, China; ^2^ Department of Translational Medicine, Scientific Research System, Geneplus -Beijing Institute, Beijing, China

**Keywords:** cell senescence, chemoresistance, ovarian cancer, organoid, TCGA

## Abstract

**Background:**

Ovarian cancer (OC) is a malignant tumor associated with poor prognosis owing to its susceptibility to chemoresistance. Cellular senescence, an irreversible biological state, is intricately linked to chemoresistance in cancer treatment. We developed a senescence-related gene signature for prognostic prediction and evaluated personalized treatment in patients with OC.

**Methods:**

We acquired the clinical and RNA-seq data of OC patients from The Cancer Genome Atlas and identified a senescence-related prognostic gene set through differential and cox regression analysis in distinct chemotherapy response groups. A prognostic senescence-related signature was developed and validated by OC patient-derived-organoids (PDOs). We leveraged gene set enrichment analysis (GSEA) and ESTIMATE to unravel the potential functions and immune landscape of the model. Moreover, we explored the correlation between risk scores and potential chemotherapeutic agents. After confirming the congruence between organoids and tumor tissues through immunohistochemistry, we measured the IC_50_ of cisplatin in PDOs using the ATP activity assay, categorized by resistance and sensitivity to the drug. We also investigated the expression patterns of model genes across different groups.

**Results:**

We got 2740 differentially expressed genes between two chemotherapy response groups including 43 senescence-related genes. Model prognostic genes were yielded through univariate cox analysis, and multifactorial cox analysis. Our work culminated in a senescence-related prognostic model based on the expression of SGK1 and VEGFA. Simultaneously, we successfully constructed and propagated three OC PDOs for drug screening. PCR and WB from PDOs affirmed consistent expression trends as those of our model genes derived from comprehensive data analysis. Specifically, SGK1 exhibited heightened expression in cisplatin-resistant OC organoids, while VEGFA manifested elevated expression in the sensitive group (*P*<0.05). Intriguingly, GSEA results unveiled the enrichment of model genes in the PPAR signaling pathway, pivotal regulator in chemoresistance and tumorigenesis. This revelation prompted the identification of potential beneficial drugs for patients with a high-risk score, including gemcitabine, dabrafenib, epirubicin, oxaliplatin, olaparib, teniposide, ribociclib, topotecan, venetoclax.

**Conclusion:**

Through the formulation of a senescence-related signature comprising SGK1 and VEGFA, we established a promising tool for prognosticating chemotherapy reactions, predicting outcomes, and steering therapeutic strategies. Patients with high VEGFA and low SGK1 expression levels exhibit heightened sensitivity to chemotherapy.

## 1 Introduction

Ovarian cancer (OC) is the primary contributor to gynecologic carcinoma worldwide. The first-line OC treatments, outlined by the NCCN guidelines, encompass comprehensive debulking surgery and platinum-based chemotherapy. In advanced stages, adjunctive antiangiogenic agents are recommended (1). While chemotherapy remains pivotal in OC treatment, about 70%-80% of patients experience relapse after treatment eventually culminating in chemotherapy resistance (2, 3).

Cellular senescence is an irreversible biological state in which cells lose their ability to proliferate and transition from the cell cycle into a relatively stable state, an indispensable mechanism for tumor suppression (4). Chemotherapy-induced senescence (CIS), represents a subtype of cellular senescence, triggered by platinum-based chemotherapy, such as cisplatin (5). Eluding CIS might serve as a plausible explanation for drug resistance (6). It has been observed that CIS cells can break free from their arrested state, re-enter the cell cycle, and exhibit significantly elevated tumor initiation potential (7). In OC, both spontaneous and drug-induced senescent cells contribute to cancer progression (8). Leveraging death receptor 5 (DR5)-selective agonists to augment treatment-induced apoptosis in senescent cancer cells may impede tumor progression (9). Consequently, prognosticating patients susceptible to senescence-induced chemotherapy resistance could hold the key to improving OC outcomes.

Organoids represent 3D multicellular structures akin to organs, enabling more precise replication of microenvironments for disease modeling, drug development, regenerative medicine, toxicology research, and personalized medicine. In 2009, Sato’s team achieved successful cultivation of the first organoid in the small intestine that can undergo long-term proliferation and passage, marking the beginning of contemporary organoid research (10). In 2017, Soragni et al. successfully constructed OC organoids and revealed the potential therapeutic effects of ReACp53 through high-throughput screening (11). Phan, meanwhile, suggested the utility of high-throughput drug screening based on organoid technology in OC treatment (12).

In our study, we performed extensive data analysis to ascertain senescence-related differential expression genes in patients with OC, distinguishing between platinum-resistant and platinum-sensitive groups. Subsequently, we formulated a predictive signature and validated its efficacy by assessing the expression of senescence genes in OC patients-derived organoids (OC PDOs). Further categorizing clinical samples into resistant and sensitive groups allowed us to scrutinize the cisplatin sensitivity and verify the prognostic potential of the signature, thereby paving the way for more personalized treatments.

## 2 Materials and methods

### 2.1 Collection of data and tumor tissues

We initiated our study by sourcing clinical information and RNA-seq profiles of patients with OC from TCGA database (https://www.cancer.gov/). We retrieved senescence-related genes from the GeneCards database (https://www.genecards.org). Single-cell RNA-seq data were acquired from GSE154600 (https://www.ncbi.nlm.nih.gov/geo/query/acc.cgi?acc=GSE154600). Tumor tissues were procured from patients with OC who underwent primary resection without prior chemotherapy, confirmed through pathology at the Department of Gynecology, Clinical Oncology School of Fujian Medical University, Fujian Cancer Hospital. The study secured approval from the ethical committee of Fujian Cancer Hospital (K2022-052-01). Informed consent was obtained from all participants, apprised of the research objectives.

### 2.2 DEGs of senescence in platinum-resistant and sensitive groups

The analysis encompassed 2740 differentially-expressed genes (DEGs) derived from the examination of raw count RNA-seq data in 197 patients with platinum sensitivity and 90 patients with platinum resistance to OC from TCGA using R package “DEseq2”. Employing Hallmark enrichment analysis, we scrutinized mechanisms or pathways with potential relevance to chemoresistance. After standardization of data by vst function in R package “DEseq2”, 18 prognostic senescence-related DEGs were extracted through univariate Cox analysis, and protein-protein interaction (PPI) was evaluated using the STRING database (https://version-11-5.string-db.org/ ).

### 2.3 Construction of the risk model

Through multivariate Cox regression analysis, we identified three independent prognostic-related genes. The normality of the expression data was verified using Kolmogorov-Smirnov test and visualized by QQ plots. To test the significance of differences between the two groups, we performed Student’s t-test for normally distributed data, and Wilcoxon rank sum test for non-normally distributed data. Subsequently, we constructed a prognosis model following the formula: risk score=SGK1*coef (SGK1)-VEGFA*coef (VEGFA). Patients were categorized into high- and low-risk groups based on the median score value, and the prognosis for these groups was scrutinized. We utilized the “survival” R package to execute Kaplan–Meier (K-M) analysis, probing for the survival differences between OC patients in the low- and high-risk groups. Based on the somatic mutation data from TCGA, we conducted gene mutation through “maftools” package. Single-cell RNA-seq data was integrated by “Seurat” and “SingleR” R packages. We normalized data with Log Normalize method, then utilized t-distributed stochastic neighbor embedding (t-SNE) via the “RunTSNE” function to cluster and visualize cell populations. Finally, we explored the expression of these two genes across various cell types.

### 2.4 Immune infiltration landscape and chemotherapy sensitivity analysis

To estimate the proportion of 22 immune cell types between the low- and high-risk groups, we employed the CIBERSORT algorithm (13). The R package “ESTIMATE” was employed to evaluate stromal, immune, and tumor purity scores of OC, based on the proportion of immune and stromal cells. We forecasted the chemotherapy response of commonly used drugs for OC patients through the Genomics of Drug Sensitivity in Cancer (GDSC; https://www.cancerrxgene.org/) database. The half-maximal inhibitory concentration (IC_50_) was determined using the “pRRophetic” package to assess chemotherapy response (14).

### 2.5 Human ovarian cancer organoid establishment, culture, and verification

**Figure 1:**
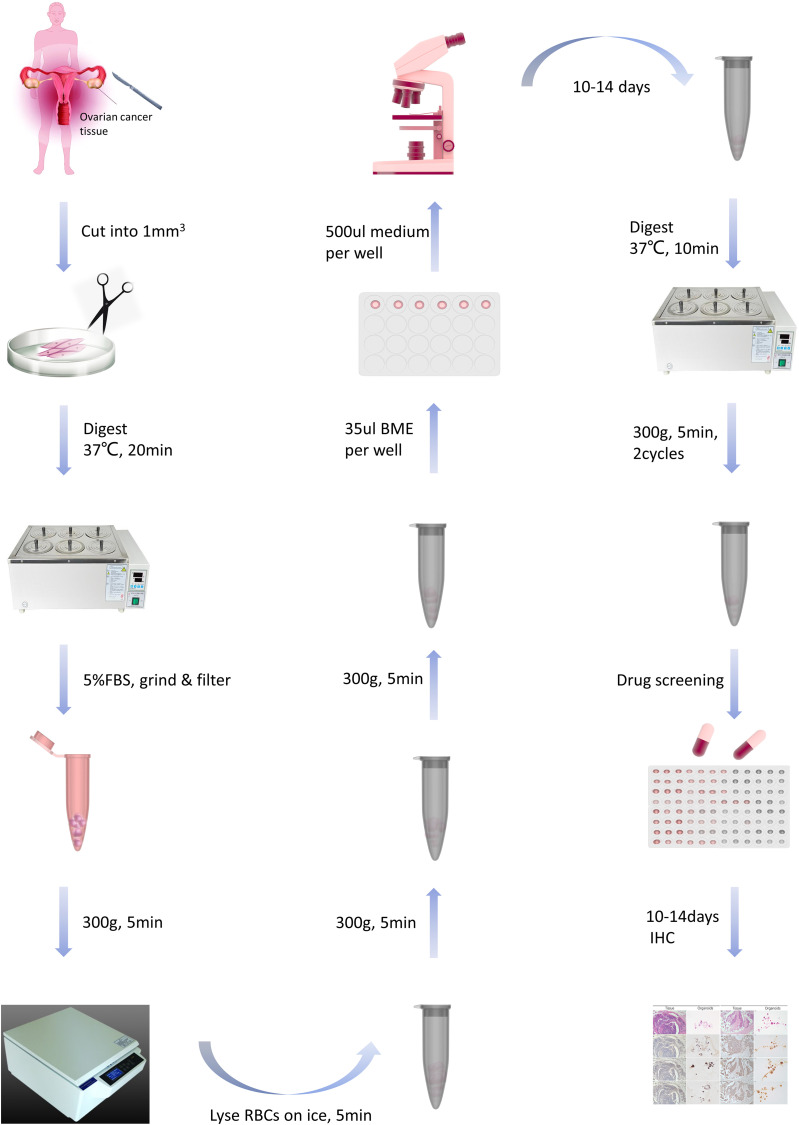
The flowchart for constructing and passaging ovarian cancer organoids.

Specimens, sized 1- 2 cm^3^, were minced into 1-mm^3^ fragments and incubated in a tissue digestion solution (K601003; Tumor Tissue Digestion Solution; bioGenous™) at 37°C for 20-40 minutes. Digestion duration was determined through microscopic observations of cells dissociating into 10- to 20-µm small clusters. The suspension was then filtered through a 100-µm nylon cell strainer and centrifuged at 300 g for 5 minutes. After lysing red blood cells using the Red Blood Cell Lysis Solution (E238010; bioGenous™) and centrifuging again to wash the cell pellet with DMEM (Gibco), a portion of the suspension was mixed with organoid cryopreservation medium (E238023; bioGenous™). The mixture was then placed in a programmed cooling box at -80°C for 24 hours and subsequently stored in liquid nitrogen. Approximately 2000-5000 cells per well were mixed with 35µL basement membrane extract (BME; bioGenous™) and seeded into pre-warmed 24-well plates. Following BME solidification, each well was incubated with 500 µL human OC organoid medium (K2168-OC; bioGenous™) at 37°C. The medium was replenished every 2-3 days. Capture images every 3 days with a microscope (TS2; Nikon) and a camera (SYA-C20). Organoids were passaged approximately every 14 days by dissociation with an organoid dissociation solution (E238001; bioGenous™) for 10 minutes at 37°C. The success rate for establishing OC PDOs was 50%. After organoids were propagated to the first generation, immunohistochemistry was performed to validate whether their origin was consistent with tumor tissue. The process is outlined in **Figure 1**.

### 2.6 ATP quantification cell viability assay

For organoid construction, the previously mentioned cell suspension was counted using a cell counter. Approximately 2500 cells per well were placed in a 96-wells 3D cell culture plate (Cat.NO: HCKB-1196UA; HonrayMed™) with 200 µL of human OC organoid isolation medium (K2168-OC; bioGenous™) at 37°C for 2-4 days. Subsequently, the medium was replenished with a medium mixture containing varying concentrations of cisplatin (catalog no. C2210000; Sigma): 0, 0.78, 1.56, 3.12, 6.25, 12.5, 25, 50, and 100 µM.) This incubation persisted for 4 days. ATP quantification was performed to determine sensitivity to cisplatin by assessing the inhibition rate of organoid activity. IC_50_, IC_90,_ and peak plasma concentration (PPC) were the evaluation metrics used to ascertain whether or not the organoids exhibited resistance to cisplatin. Cases where IC_50_>25% PPC and IC_90_>100% PPC were classified as resistant, while those with IC_50_<25% PPC and IC_90_< or > 100% PPC, or IC_50_>25% PPC and IC_90_<100% PPC, were deemed sensitive (15).

### 2.7 Quantitative real-time polymerase chain reaction and western blot

Total RNA extraction for human OC organoids in the sensitive and resistant groups was performed following the instructions of the RNA extraction kit (LS1040; Promega). cDNAs were synthesized through reverse transcription (GoScript™ Reverse Transcription Mix, Oligo(dT) A2790; Promega). Quantitative polymerase chain reaction (Q-PCR) was performed using the SYBR Green Master Kit (Roche), with mRNA expression levels normalized to GAPDH. The primer sequences are provided as follows: SGK1-F: aaacacagctgaaatgtacgac; SGK1-R: ttggttaaaagggggagtaatc; VEGFA-F: atcgagtacatcttcaagccat; VEGFA-R: gtgaggtttgatccgcataatc; GAPDH-F: tgtgggcatcaatggatttgg; GAPDH-R: acaccatgtattccgggtcaat. After culturing P1 generation organoids for 14 days, collected organoids and lysed with RIPA buffer (Epizyme Biotech, PC101), The BCA protein assay kit (Epizyme Biotech, ZJ101) was used to measure protein concentration. The proteins were separated on SDS-PAGE gels and transferred to polyvinylidene fluoride (Millipore, IPVH00010, 0.45µm) and then blocked with 5% milk for 1 h at RT and immunoblotted with primary antibodies at 4°C overnight: GAPDH (cell signaling technology, 2118, 1:1000 dilution), VEGFA (Proteintech, 66828-1-Ig, 1:1000 dilution), SGK1 (Proteintech, 28454-1-AP, 1:1000 dilution). Finally, the membranes were incubated with secondary antibodies (abcam, ab205718, ab205719, 1:10000 dilution) and visualized by imaging systems.

### 2.8 Statistical analysis

Statistical analyses were carried out using Perl scripts (v5.30.0), R software (v.4.1.0), and GraphPad Prism (v8.0.2). Kaplan-Meier (K-M) analysis was employed to assess survival differences. Differential functions were analyzed using the Wilcoxon rank-sum test between two groups. All experiments were repeated in triplicate. The data were expressed as mean ± standard. *P* < 0.05 was considered as statistical significance.

## 3 Results

### 3.1 Validation of senescence-related genes in the platinum-sensitive and resistant OC groups

**Figure 2:**
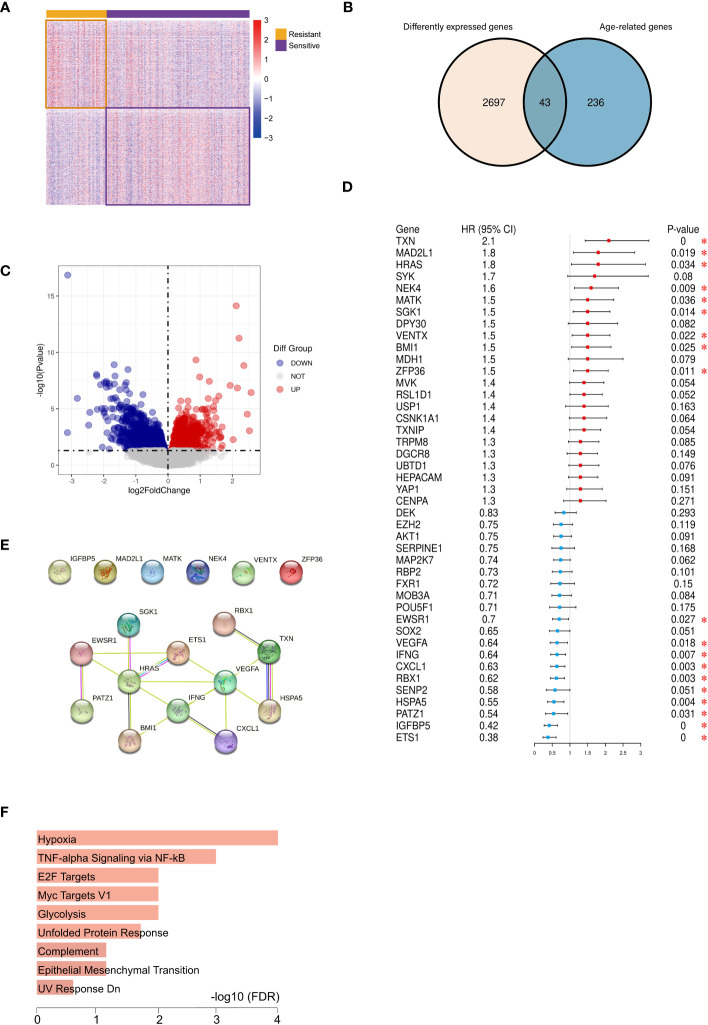
Identification of 12 vital differentially expressed senescence genes in ovarian cancer. Heatmap **(A)** and volcano plot **(C)** of DEGs between the platinum-resistant and sensitive OC patients based on TCGA. **(B)** Venn diagram of ageing-genes and DEGs, **(D)** Univariate Cox analysis of the aging-related DEGs expression. **(E)** PPI network illustrated the relationship among the 12 DEGs. **(F)** Hallmark enrichment analysis of the aging-related DEGs. (*P < 0.05).

Our investigation harnessed clinical data from the TCGA database, yielding 197 cases in the platinum-sensitive group and 90 cases in the drug-resistant group. Clinical pathological parameters were shown in **Supplementary Table 1**. Subsequently, RNA-seq data for these cases were acquired and subjected to analysis, resulting in the identification of 2740 DEGs (|log2FC|>0 and *P*<0.05), including 1373 upregulated and 1367 downregulated genes, which were then visualized through heat-maps and volcano plots (**Figures 2A, C**). We further extracted 43 aging-related DEGs by intersecting 279 aging-associated genes with the 2740 DEGs (**Figure 2B**). Employing univariate Cox analysis, we identified 18 genes exhibiting significant correlations with prognosis, including 9 genes with negative associations and 9 with positive correlations (**Figure 2D**).

These 18 prognosis-linked genes were used to construct PPI networks, from which 12 pivotal genes formed a central network marked by interactions (**Figure 2E**). Functional enrichment analysis indicated their enrichment in pathways associated with hypoxia, inflammation, and glycolysis processes closely intertwined with cellular aging (**Figure 2F**).

### 3.2 Construction of a senescence-related gene signature in platinum-resistant and -sensitive OC groups

**Figure 3:**
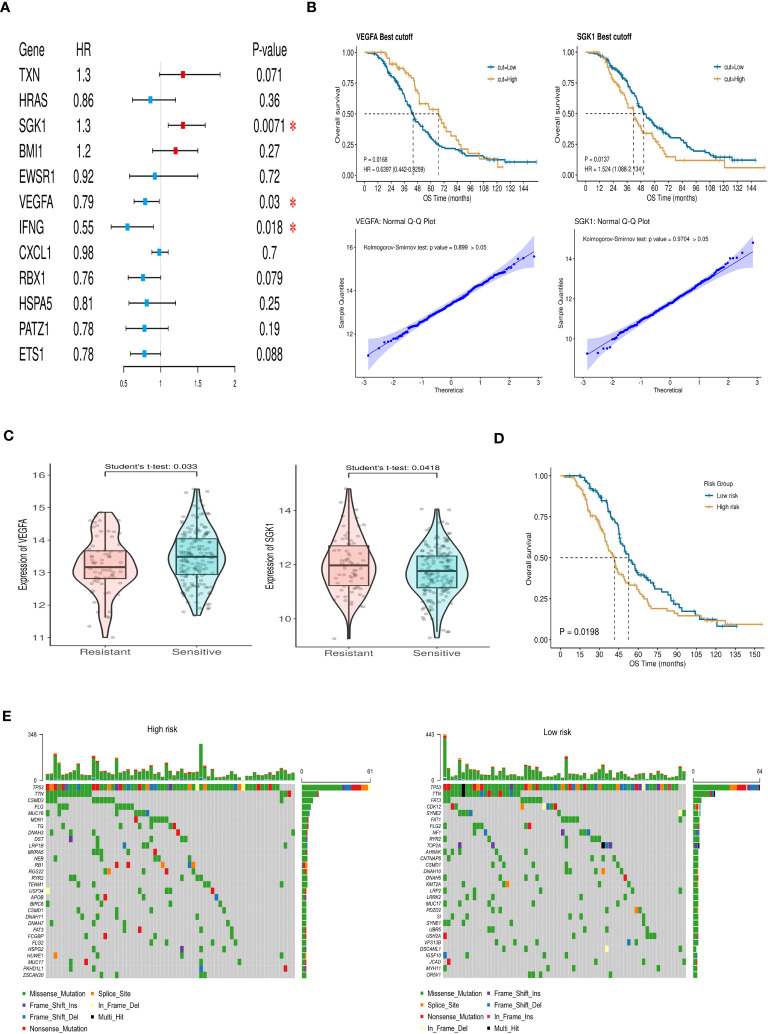
Construction and verification of the prognostic index. **(A)** Forest plot of aging-related DEGs via multivariate Cox regression analysis. **(B)** The survival ability of OC patients with high and low expression level of VEGFA (*P*=0.0168) and SGK1 (*P*=0.0137). Q-Q plot of VEGFA and SGK1 to test the normal distribution of data. **(C)** The expression of VEGFA and SGK1 in resistant and sensitive groups. **(D)** Differences in overall survival between high- and low-risk groups (*P*=0.0198). **(E)** Mutation waterfall maps show the gene mutation differences in high- and low-risk groups. (* *P*<0.05).

**Figure 4:**
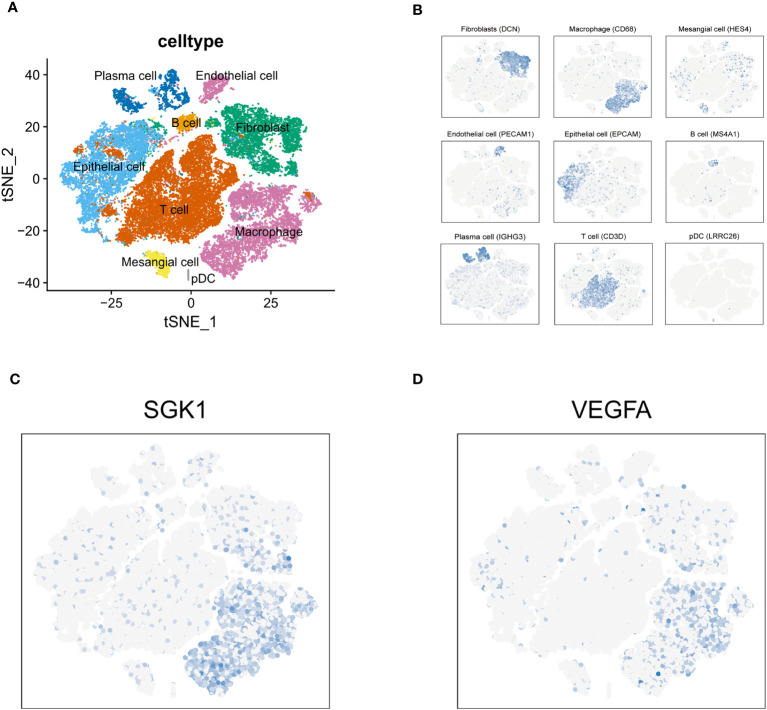
Tumor immune microenvironment. **(A)** t-SNE plot visualized 9 cell subtypes in OC patients. **(B)** The annotation diagram of different cell types. **(C, D)** The distribution of SGK1 and VEGFA expression in all cell types.

Through multivariate Cox regression analysis on these 12 core genes, we obtained 3 genes significantly link to prognosis, depicted in **Figure 3A**. Distinct survival ability of OC patients with different expression level of IFNG, SGK1 and VEGFA were discerned (*P*<0.05) (**Supplementary Figure 1A**) (**Figure 3B**). The normality of the expression data was verified using Kolmogorov-Smirnov test and visualized by QQ plots. The expression of SGK1 and VEGFA was verified to be normally distributed (**Figure 3B**), whereas IFNG was not (**Supplementary Figure 1B**). Student’s t-test was used to exam the differences in expression levels between the platinum-resistant and sensitive groups for SGK1 and VEGFA, and IFNG was using the Wilcoxon rank sum test (**Supplementary Figure 1C**). Notably, SGK1 and VEGFA demonstrated significantly different expression (*P*<0.05), while IFNG presented no significant expression differences between two groups (**Figure 3C**). Therefore, we included SGK1 and VEGFA in our risk model construction following the formula, risk score=SGK1*coef (SGK1)-VEGFA*coef (VEGFA) (coef values were in **Supplementary Table 2**). Segregation of patients based on risk scores yielded the high- and low-risk groups, with those in the high-risk category facing a worse prognosis (*P*<0.05, **Figure 3D**). Gene mutation waterfall plots for both groups are showcased in **Figure 3E**. In the GSE154600 dataset, there are 9 cell types marked mainly including cancer, immune, and stromal cells, and a t-SNE plot visualized cell subtypes in patients with OC (**Figure 4A**), with subtype annotations provided in **Figure 4B**. Both SGK1 and VEGFA exhibited increased expression in macrophages, suggesting a correlation between these risk genes and macrophage dysregulation in OC (**Figures 4C, D**).

### 3.3 GSEA and immune microenvironment status

**Figure 5:**
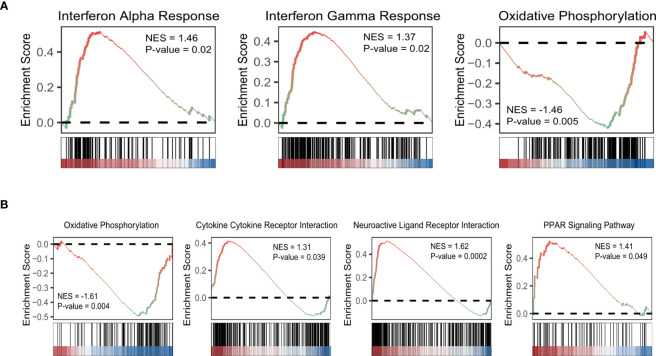
GSEA of aging-related DEGs based on hallmark gene sets **(A)** and KEGG database **(B)**. (NES: normalized enriched score).

**Figure 6:**
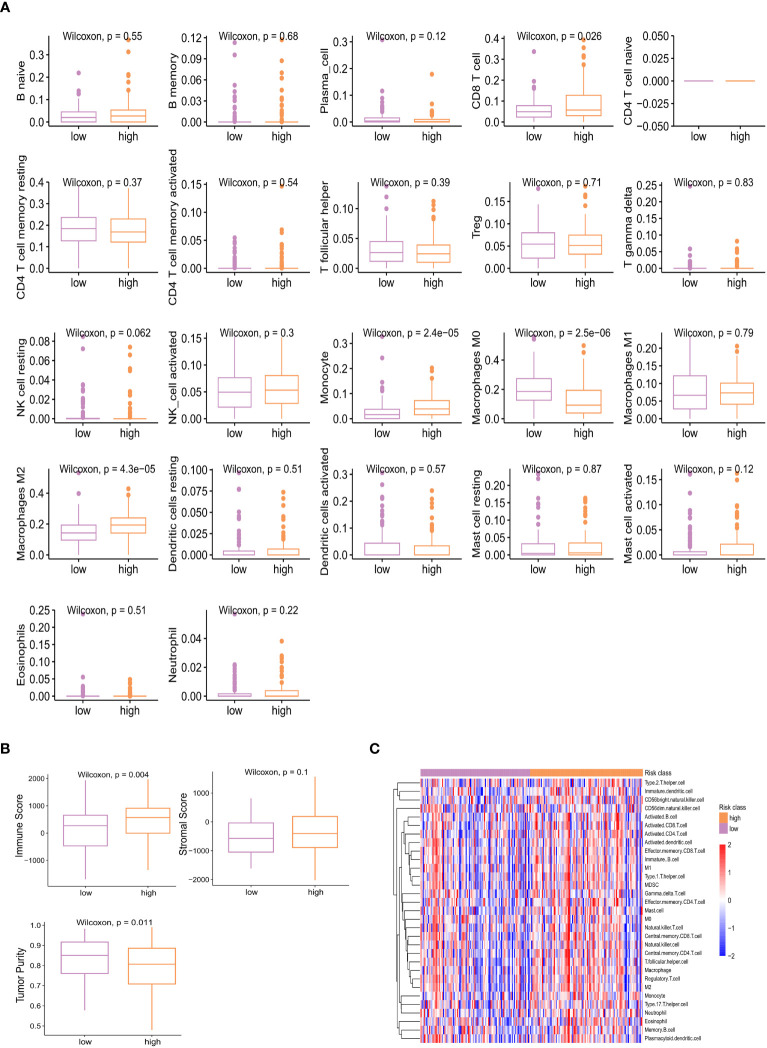
Different proportion of 22 immune cells between high- and low-risk groups analyzed by CIBERSORT algorithm **(A)** and heatmap **(C)**. **(B)** Estimate analysis of immune, stromal, and tumor purity score in high- and low-risk group.

Based on KEGG database, genes with higher expression levels in resistant group were involved in the peroxisome proliferator-activated receptor (PPAR) signaling pathway, Cytokine Receptor Interaction pathway, and Neuroactive Ligand Receptor Interaction pathway (*P*<0.05), the same trend was found in Interferon Alpha/Gamma Response pathway based on hallmark gene sets. While elevated involvement of oxidative phosphorylation pathway was found in genes highly expressed in sensitive group (*P*<0.01; **Figures 5A, B**). We carried out the CIBERSORT algorithm to analyze the proportion of the 22 immunocyte. Discrepancies in the microenvironment between high- and low-risk groups primarily centered around CD8^+^ T cell, monocytes, and macrophages (**Figure 6A**). Estimate analysis depicted correlations between immune, stromal and tumor purity scores with high- and low-risk scores (**Figure 6B**). **Figure 6C** illustrated the heatmap of differences in infiltrating immune cells between the two groups.

### 3.4 Prediction of potential drugs

**Figure 7:**
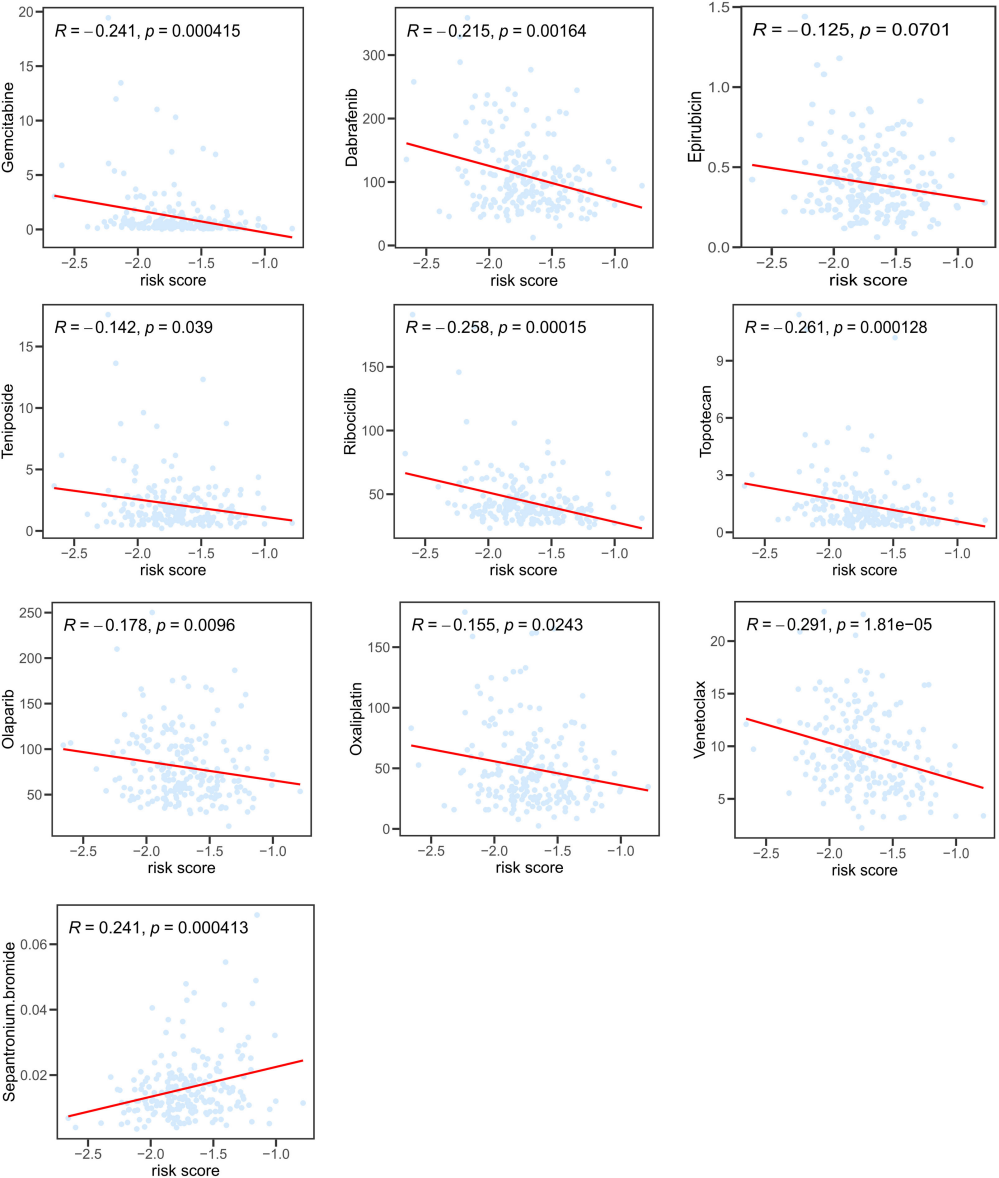
Correlation between risk score and IC_50_ of potential chemotherapeutics predicted by the “pRRophetic” package.

We further investigated the correlations between the IC_50_ of potential target drugs and risk scores. The smaller IC_50_ presented, the more sensitive patients were to drugs. Patients with high risk appeared to benefit from drugs such as gemcitabine, dabrafenib, epirubicin, oxaliplatin, olaparib, teniposide, ribociclib, topotecan, and venetoclax (**Figure 7**), while others seemed more beneficial for patients in the low-risk group.

### 3.5 Cultivation and verification in human ovarian cancer organoids

**Figure 8:**
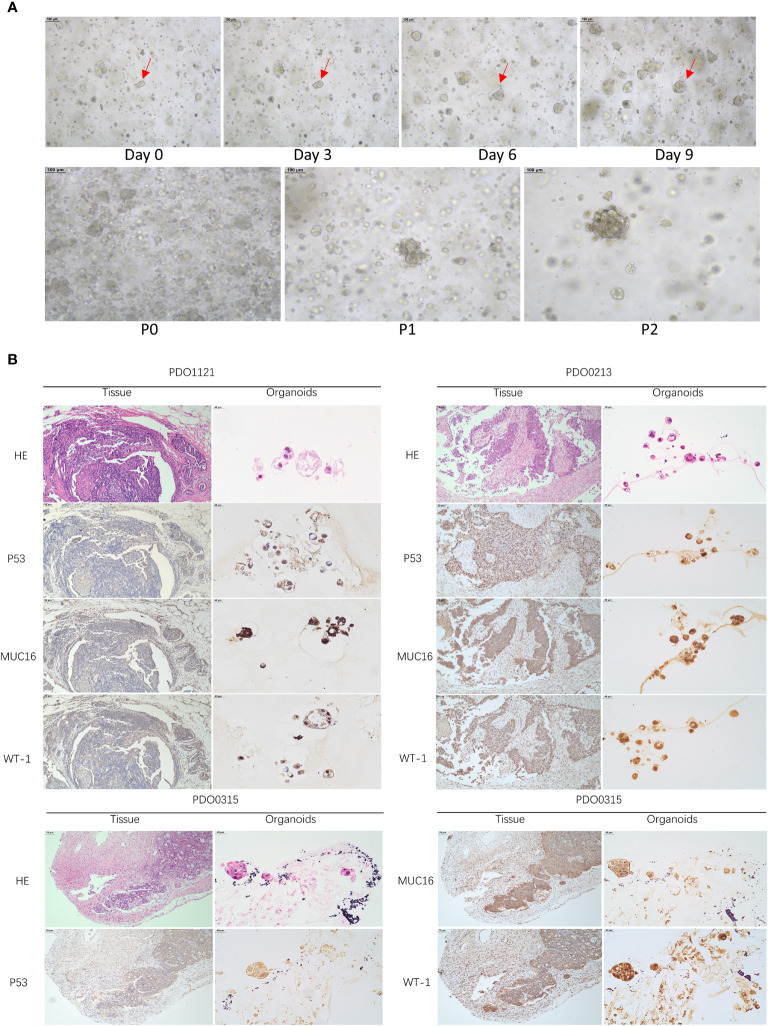
**(A)** The growth states of organoids recorded. (Scale bar = 100 µm) (The red arrow indicates the same organoid) **(B)** HE and IHC staining of P53, MUC16, WT-1 in OC tissues and organoids (Scale bar for tissue = 70 µm, scale bar for organoid = 40 µm).

**Figure 9:**
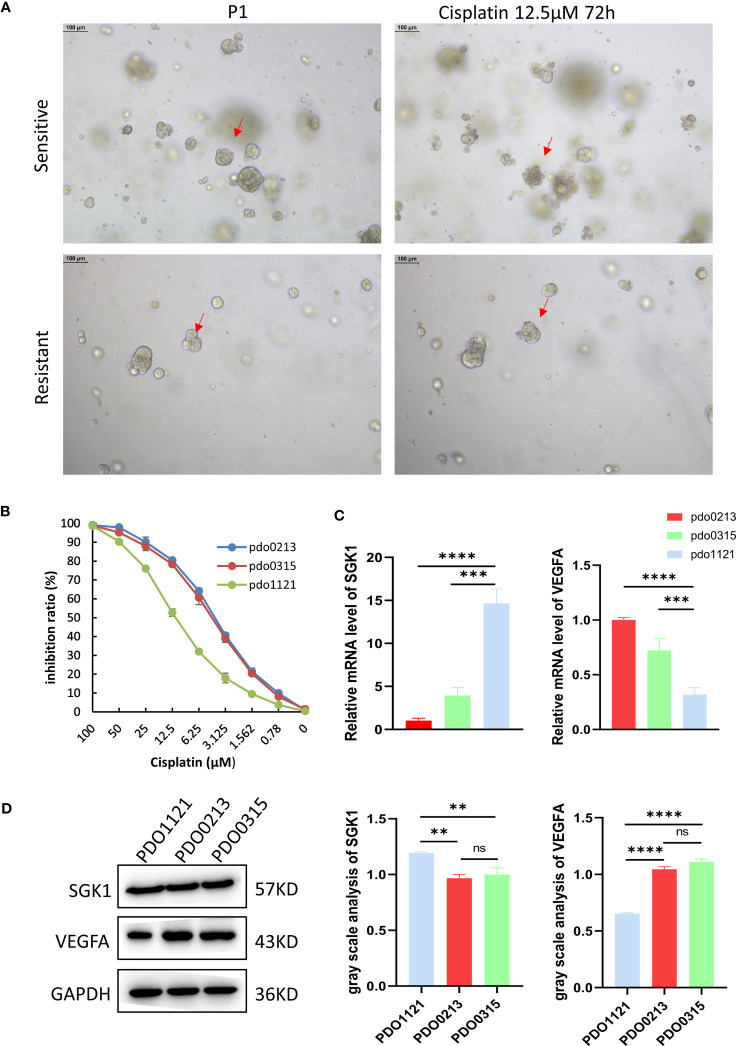
**(A)** The different state of organoids in two groups after culturing 72 hours with 12.5 μM cisplatin. The red arrows indicate organoid changes. **(B)** The inhibition ratio plot of organoids under different concentrations cisplatin. Data presented as mean ± SD. **(C)** Relative expression level of SGK1 and VEGFA in organoids. Data presented as the mean ± SEM, one-way ANOVA was used for statistical calculation, n=3 independent experiments. **(D)** Western blot and gray values of SGK1 and VEGFA expression in PDOs. (Scale bar = 100 µm, ns: No significance **P < 0.01. ***P < 0.001, ****P < 0.0001).

Our study successfully obtained and cultured three OC samples into organoids, visually documented through growth progression as indicated by the red arrows in **Figure 8A**. The development of organoids from P0 to P2 showed in **Figure 8A** (Scale bar = 100 µm). Furthermore, patients’ clinical data are summarized in **Table 1**. Immunohistochemical testing of p53, WT-1, and MUC16 in the organoids, the routine marker in diagnosing OC, exhibited striking concordance with tissue expression, (**Figure 8B**, Scale bar for tissue = 70 µm, scale bar for organoid = 40 µm), affirming the homology between the organoids and the corresponding tumor tissue. We exposed organoids from the P1 generation to cisplatin diluted with medium across varying concentrations and captured their growth state after 72 hours. **Figure 9A** showed the representative growth status changes under 12.5 µM cisplatin medium in drug-resistant and sensitive PDOs (Scale bar = 100 µm). Notably, the sensitive group’s organoids exhibited shrinkage, rupture, and loss of original structure following cisplatin addition. By contrast, organoids in the resistant group displayed only mild shrinkage. The dose-response curve reflecting drug inhibition rates on the organoids is illustrated in **Figure 9B**. Organoids were categorized into cisplatin-resistant and -sensitive groups based on IC_50_ and IC_90_. Specifically, PDO1121 was recognized as cisplatin-resistant sample, whereas PDO0213, and PDO0315 were designated as the cisplatin-sensitive ones. In these groups, VEGFA exhibited low expression in cisplatin-resistant organoids, while SGK1 was prominently expressed (**Figure 9C**). **Figure 9D** illustrated the protein expression and the gray scale analysis of SGK1 and VEGFA in OC PDOs. SGK1 were higher expressed in PDO1121 and VEGFA were higher in PDO0213 and PDO0315.

**Table 1:** Clinical information of patients for culturing organoids.

Organoid	Age(years)	Tumor type	Presentation	FIGO	Treatment	CA125 returned to normal level (chemotherapy cycles)
PDO1121	51	EOC	Primary	IIIB	TC	2
PDO0213	39	EOC	Primary	IVB	TC	2
PDO0315	62	EOC	Primary	IVB	TC	*

(TC: Paclitaxel+ Carboplatin, * As of the time of paper writing, the patient has received 3 cycles of chemotherapy without CA125 normal).

## 4 Discussion

Cellular senescence, instigated by DNA damage response, unfolds distinct characteristics: an irreversible growth halt; augmented lysosomal activity (16), amplified damage response signaling pathways; the emergence of macromolecular damage (17), and a senescence-associated secretory phenotype (SASP) (18, 19). Senescence operates as a dual-edged sword. In normal tissue, it can impair repair and regeneration, accentuating aging,as well as maintain normal tissue homeostasis by immune-mediated clearance (20). In tumor tissue, it serves as a robust anti-tumor mechanism, thwarting the proliferation of cancerous cells and the genetic transfer of damaged cells on the one hand (21), but also promotes cellular reprogramming into a stem-like state, resulting in drug-resistant and invasive clones on the other hand (22). Contemporary studies spotlight chemotherapy and radiation as catalysts for treatment-induced senescence (TIS) within tumor cells (23). And CIS could interact with tumor micro environment (TME). In breast cancer it is confirmed CIS could change TME and increase the aggressiveness through the CXCL11 signaling pathway (24). Nicolas Adele et al. also revealed that the senescence of inflammatory cancer-associated fibroblasts (iCAFs) induced by chemoradiotherapy was tightly correlated with poor prognosis in rectal cancer (25). Meanwhile, cellular senescence might induce EMT and drug resistance through the PI3K/AKT pathway in colorectal cancer (26). Additionally, in lung cancer, TIS-induced cisplatin resistance may be pivotal to autophagy and hypoxia (27). The study of OC manifests TIS within stromal cells and exhibits SASP through cancer-associated fibroblasts (CAFs), which underlies resistance of PARP inhibitors (28). Hence, the study of cellular senescence-related therapeutic targets may help to overcome tumor drug resistance. Taking death receptor 5 (DR5) as an example, the activation of DR5 may impede tumor progression, not only through the senescent cells but also non-senescent cells adjacent by bystander effect (29). Furthermore, a novel nanoplatform has been studied targeting tumor necrosis factor-related apoptosis-inducing ligand (TRAIL) to further strengthen DR5-induced apoptosis, which provides a promising approach to clinically overcome tumor resistance (30).

In our investigation, a novel senescence-related gene signature sourced from patients with platinum-resistant and platinum-sensitive OC, featuring genes such as SGK1 and VEGFA, was constructed with potential prognostic predictive value. The biological functions of these model genes are listed in **Table 2**. Patients were classified into low- and high-risk cohorts based on the median scores. Notably, patients in the low-risk group displayed superior prognoses, while those at higher risk had a significantly worse prognosis. SGK1 exhibited elevated expression, while VEGFA presented a low expression trend in the platinum-resistant group with a poor prognosis. We speculate that this may be related to poor angiogenesis in the platinum-resistant OC patients and the chemotherapeutic agents are unable to reach the tumor site. The low expression of VEGFA may also be strongly associated with the acquisition of resistance to bevacizumab leading to poorer outcomes in patients with platinum-resistant (39). Through the construction of OC PDOs, the credibility of this predictive signature was validated. Nonetheless, further inquiry remains imperative to elucidate the precise effect of these senescence-related genes on the prognosis of patients with OC.

**Table 2:** Biological functions of model genes.

Gene	Physiological functions	Tumor immune microenvironment	Roles in OC	Other cancers
SGK1	glycolysis, angiogenesis, immune regulation, cell migration, tissue fibrosis and calcification (31, 32)	promotes CD8 T cell exhaustion and anti-PD1 reaction (33)	induce paclitaxel resistance in OC cells (34)	Lung adenocarcinoma (35) HCC (33)
VEGFA	angiogenesis, vascular maintenance and leakage (36, 37)	inhibit DC maturation and T-cell infiltration (38)	low expression improve resistance to bevacizumab (39)	HCC (40) Lymphoma (41)

OS, overall survival; PFS, progression-free survival; DC, dendritic cell; HCC, hepatocellular carcinoma

The GSEA unveiled significant enrichment of high expressed genes in chemotherapy-resistant cohorts in the PPAR signaling pathway. PPAR encompasses three isoforms: PPARα, PPARβ/δ, and PPARγ-a nuclear receptor family pivotal in regulating and transcribing target genes, energy metabolism, cellular dynamics, inflammation, and carcinogenesis (42, 43). The interplay between the PPAR pathway and chemoresistance has garnered attention across diverse cancers, including diffuse large B-cell lymphoma (DLBCL) (44), breast cancer (45), hepatocellular carcinoma (HCC) (46), non-small cell lung cancer (NSCLC) (47), and OC (48). In addition, activation of the PPAR gamma signaling pathway can balance the release of inflammatory and anti-inflammatory cytokines, orchestrating a pre-malignant microenvironment that promotes cell senescence, and alleviating tumor burdens (49). In endometrial carcinoma, inhibition of PARR through Bcl-2/Caspase3 pathways could hinder apoptosis, fostering carcinogenesis (50). In our investigation, the activation of the PPAR pathway was correlated with poor prognosis in OC patients.

To further validate the prognostic potential of our risk model, we constructed the OC PDOs. Organoids are intricate 3D multicellular structures that distinguish themselves from patient-derived xenograft (PDX), which entail drawbacks such as high costs and extended durations, as well as 2D cell lines that suffer from limitations such as the absence of cell morphology, original tumor tissue structure, and intercellular interactions. Tumor organoids possess distinctive attributes: they adeptly mimic microenvironments, foster *in vitro* miniature tumor formations, and closely mirror the differentiation and expression characteristics of the original tumor tissue. This renders them highly suitable for investigating tissue responses to drugs, injuries, or mutations, conducting drug candidate screenings, and enabling precision clinical treatments (51). Phan et al. previously indicated the potential application of high-throughput drug screening based on organoid technology in OC treatment (12). In our study, we developed three human OC organoids, predicted patient responses to cisplatin treatment through ATP viability assay, and corroborated the expression of model genes (SGK1 and VEGFA) within cisplatin-resistant and cisplatin-sensitive groups. Notably, our findings aligned consistently with the analysis conducted on the TCGA database.

In this endeavor, a senescence-linked risk model was devised, underscoring the connection between model gene and PDO drug responses to cisplatin treatment. A broader sample set of clinical trials is necessary to adequately assess the clinical significance of our features. However, few patients have primary resistance in the clinical setting, while platinum-resistant patients tend to relapse six months after initial treatment, with fewer opportunities for surgery and difficult access to specimens. Due to the difficulty of obtaining enough platinum-resistant tissue to culture organoids, there are limitations to our organoid validation cohort. We plan to conduct further fundamental experiments to explore the complex mechanisms of senescence-related genes in OC’s chemoresistance.

## 5 Conclusion

Through a holistic analysis and rigorous *in vitro* validation, our study conceived a senescence-related gene signature (SGK1 and VEGFA) in OC. Our model demonstrated the potential to forecast chemotherapy outcomes and prognosis, guiding therapeutic interventions.

## Data availability statement

The RNA sequencing profiles and clinical information of OC patients are able to be gained from The Cancer Genome Atlas (TCGA) (https://toil.xenahubs.net). Senescence-related gene set was retrieved from the GeneCards database (https://www.genecards.org). Single-cell RNA-seq data was downloaded from GSE154600. Further inquiries can be directed to the corresponding author.

## Ethics statement

The studies involving humans were approved by ethical committee of Fujian Cancer Hospital (K2022-052-01). The studies were conducted in accordance with the local legislation and institutional requirements. The participants provided their written informed consent to participate in this study.

## Author contributions

XC: Writing – original draft, Writing – review & editing. YL: Writing – review & editing. JZ: Software, Visualization, Writing – original draft. LL: Methodology, Writing – original draft. ZJ: Writing – review & editing, Visualization. JL: Data curation, Writing – original draft. SJ: Data curation, Investigation, Writing – original draft. XL: Resources, Writing – original draft. YS: Funding acquisition, Project administration, Supervision, Writing – review & editing.

## References

[1] Armstrong D Alvarez R Backes F Bakkum-Gamez J Barroilhet L Behbakht K. NCCN guidelines® Insights: ovarian cancer, version 3.2022. J Natl Compr Cancer Network JNCCN (2022) 20(9):972–80. doi: 10.6004/jnccn.2022.0047 36075393

[2] Alatise KL Alexander-bryant GS. A mechanisms of drug resistance in ovarian cancer and associated gene targets. Cancers (2022) 14(24):6246. doi: 10.3390/cancers14246246 36551731 PMC9777152

[3] Foley OW R-HJ del Carmen MG. Recurrent epithelial ovarian cancer: an update on treatment. Oncol (Williston Park) (2013) 27(4):288–98. 23781692

[4] PJ H. Senescence as an anticancer mechanism. J Clin Onco (2007) 25(14):1852–7. doi: 10.1200/JCO.2006.10.3101 17488983

[5] Nacarelli T Fukumoto T Zundell J Fatkhutdinov N Jean S Cadungog M. NAMPT inhibition suppresses cancer stem-like cells associated with therapy-induced senescence in ovarian cancer. Cancer Res (2020) 80(4):890–900. doi: 10.1158/0008-5472.CAN-19-2830 31857293 PMC7024650

[6] Guillon J Petit C Toutain B Guette C Lelièvre E Coqueret O. Chemotherapy-induced senescence, an adaptive mechanism driving resistance and tumor heterogeneity. Cell Cycle (Georgetown Tex) (2019) 18(19):2385–97. doi: 10.1080/15384101.2019.1652047 PMC6738909 31397193

[7] Saleh T T-ML Gewirtz DA. Tumor cell escape from therapy-induced senescence as a model of disease recurrence after dormancy. Cancer Res (2019) 79(6):1044–6. doi: 10.1158/0008-5472.CAN-18-3437 30803994

[8] Rutecki S Szulc P Pakuła M Uruski P Radziemski A Naumowicz E. Pro-cancerogenic effects of spontaneous and drug-induced senescence of ovarian cancer cells *in vitro* and *in vivo*: a comparative analysis. J Ovarian Res (2022) 15(1):87. doi: 10.1186/s13048-022-01023-y 35883110 PMC9317468

[9] Soto-Gamez A Wang Y Zhou X Seras L Quax W Demaria M. Enhanced extrinsic apoptosis of therapy-induced senescent cancer cells using a death receptor 5 (DR5) selective agonist. Cancer Lett (2022) 525:67–75. doi: 10.1016/j.canlet.2021.10.038 34728311

[10] Sato T Vries R Snippert H van de Wetering M Barker N Stange D. Single Lgr5 stem cells build crypt-villus structures *in vitro* without a mesenchymal niche. Nature (2009) 459(7244):262–5. doi: 10.1038/nature07935 19329995

[11] Soragni A Janzen D Johnson L Lindgren A Thai-Quynh Nguyen A Tiourin E. A Designed Inhibitor of p53 Aggregation Rescues p53 Tumor Suppression in Ovarian Carcinomas. Cancer Cell (2016) 29(1):90–103. doi: 10.1016/j.ccell.2015.12.002 26748848 PMC4733364

[12] Phan N Hong J Tofig B Mapua M Elashoff D Moatamed N. A simple high-throughput approach identifies actionable drug sensitivities in patient-derived tumor organoids. Commun Biol (2019) 2:78. doi: 10.1038/s42003-019-0305-x 30820473 PMC6389967

[13] Newman AM LC Green MR Gentles AJ Feng W Xu Y Hoang CD. Robust enumeration of cell subsets from tissue expression profiles. Nat Methods (2015) 12(5):453–7. doi: 10.1038/nmeth.3337 PMC4739640 25822800

[14] Geeleher P Cox N Huang RS. pRRophetic: an R package for prediction of clinical chemotherapeutic response from tumor gene expression levels. PLoS One (2014) 9(9):e107468. doi: 10.1371/journal.pone.0107468 25229481 PMC4167990

[15] Driehuis E Kretzschmar K Clevers H. Establishment of patient-derived cancer organoids for drug-screening applications. Nat Protoc (2020) 15(10):3380–409. doi: 10.1038/s41596-020-0379-4 32929210

[16] Lee B Han J Im J Morrone A Johung K Goodwin E. Senescence-associated beta-galactosidase is lysosomal beta-galactosidase. Aging Cell (2006) 5(2):187–95. doi: 10.1111/j.1474-9726.2006.00199.x 16626397

[17] Gorgoulis V Adams P Alimonti A Bennett D Bischof O Bishop C. Cellular senescence: defining a path forward. Cell (2019) 179(4):813–27. doi: 10.1016/j.cell.2019.10.005 31675495

[18] Kumari RJP. Mechanisms of cellular senescence: cell cycle arrest and senescence associated secretory phenotype. Front Cell Dev Biol (2021) 9:645593. doi: 10.3389/fcell.2021.645593 33855023 PMC8039141

[19] Saleh T Tyutynuk-Massey L Cudjoe E Idowu M Landry J Gewirtz D. Non-cell autonomous effects of the senescence-associated secretory phenotype in cancer therapy. Front Oncol (2018) 8:164. doi: 10.3389/fonc.2018.00164 29868482 PMC5968105

[20] Coppé J Desprez P Krtolica A Campisi J. The senescence-associated secretory phenotype: the dark side of tumor suppression. Annu Rev Pathol (2010) 5:99–118. doi: 10.1146/annurev-pathol-121808-102144 20078217 PMC4166495

[21] Wang B Kohli J Demaria M. Senescent cells in cancer therapy: friends or foes? Trends Cancer (2020) 6(10):838–57. doi: 10.1016/j.trecan.2020.05.004 32482536

[22] Chakrabarty A CS Bhattacharya R Chowdhury G. Senescence-induced chemoresistance in triple negative breast cancer and evolution-based treatment strategies. Front Oncol (2021) 11):674354. doi: 10.3389/fonc.2021.674354 34249714 PMC8264500

[23] Liu H Zhao H Sun Y. Tumor microenvironment and cellular senescence: Understanding therapeutic resistance and harnessing strategies. Semin Cancer Biol (2022) 86(Pt 3):769–81. doi: 10.1016/j.semcancer.2021.11.004 34799201

[24] Hwang HJ LY Kang D Lee HC Seo HR Ryu JK Kim YN. : Endothelial cells under therapy-induced senescence secrete CXCL11, which increases aggressiveness of breast cancer cells. Cancer Lett (2020) 490):100–10. doi: 10.1016/j.canlet.2020.06.019 32659248

[25] Nicolas AM Pesic M Engel E Ziegler PK Diefenhardt M Kennel KB. Inflammatory fibroblasts mediate resistance to neoadjuvant therapy in rectal cancer. Cancer Cell (2022) 40(2):168–84. doi: 10.1016/j.ccell.2022.01.004 35120600

[26] Kehagias P Kindt N Krayem M Najem A Agostini G Acedo Reina E. Regorafenib induces senescence and epithelial-Mesenchymal transition in colorectal cancer to promote drug resistance. Cells (2022) 11(22):3663. doi: 10.3390/cells11223663 36429091 PMC9688587

[27] Olszewska A Borkowska A Granica M Karolczak J Zglinicki B Kieda C. Escape from cisplatin-induced senescence of hypoxic lung cancer cells can be overcome by hydroxychloroquine. Front Oncol (2021) 11:738385. doi: 10.3389/fonc.2021.738385 35127467 PMC8813758

[28] Jin P Li X Xia Y Li H Li X Yang Z. Bepotastine sensitizes ovarian cancer to PARP inhibitors through suppressing NF-κB-Triggered SASP in cancer-Associated fibroblasts. Mol Cancer Ther (2023) 22(4):447–58. doi: 10.1158/1535-7163.MCT-22-0396 36780236

[29] Wang L Jie H Jochems F Wang S Lieftink C Martinez IM. cFLIP suppression and DR5 activation sensitize senescent cancer cells to senolysis. Nat Cancer (2022) 3(11):1284–99. doi: 10.1038/s43018-022-00462-2 36414711

[30] Li F WX Wu M Guan J Liang Y Liu X Lin X. Liu J Biosynthetic cell membrane vesicles to enhance TRAIL-mediated apoptosis driven by photo-triggered oxidative stress. Biomater Sci (2022) 10(13):3547–58. doi: 10.1039/D2BM00599A 35616096

[31] Jang H Park Y Jang J. Serum and glucocorticoid-regulated kinase 1: Structure, biological functions, and its inhibitors. Front Pharmacol (2022) 13:1036844. doi: 10.3389/fphar.2022.1036844 36457711 PMC9706101

[32] Lang F Rajaxavier J Singh Y Brucker S Salker M. The enigmatic role of serum & Glucocorticoid inducible kinase 1 in the endometrium. Front Cell Dev Biol (2020) 8:556543. doi: 10.3389/fcell.2020.556543 33195190 PMC7609842

[33] Rong D Wang Y Liu L Cao H Huang T Liu H. GLIS1 intervention enhances anti-PD1 therapy for hepatocellular carcinoma by targeting SGK1-STAT3-PD1 pathway. J Immunother Cancer (2023) 11(2):e005126. doi: 10.1136/jitc-2022-005126 36787938 PMC9930610

[34] D'Antona L Dattilo V Catalogna G Scumaci D Fiumara C Musumeci F. In preclinical model of ovarian cancer, the SGK1 inhibitor SI113 counteracts the development of paclitaxel resistance and restores drug sensitivity. Trans Oncol (2019) 12(8):1045–55. doi: 10.1016/j.tranon.2019.05.008 PMC6545392 31163384

[35] Pan H Lv W Li Z Han W. SGK1 protein expression is a prognostic factor of lung adenocarcinoma that regulates cell proliferation and survival. Int J Clin Exp Pathol (2019) 12(2):391–408. 31933845 PMC6945076

[36] Apte R Chen D Ferrara N. VEGF in signaling and disease: beyond discovery and development. Cell (2019) 176(6):1248–64. doi: 10.1016/j.cell.2019.01.021 PMC6410740 30849371

[37] Fontemaggi G. Non-coding RNA regulatory networks in post-transcriptional regulation of VEGFA in cancer. IUBMB Life (2023) 75(1):30–9. doi: 10.1002/iub.2620 PMC10084289 35467790

[38] Ribatti D. Immunosuppressive effects of vascular endothelial growth factor. Oncol Lett (2022) 24(4):369. doi: 10.3892/ol.2022.13489 36238855 PMC9494354

[39] Yagi T SK Miyamoto M Shimizu A Oi Y Toda A Nakamura K. Continuous Administration of Anti-VEGFA Antibody Upregulates PAI-1 Secretion from Ovarian Cancer Cells via miR-143-3p Downregulation. Mol Cancer Res (2023) 21(10):1093–106. doi: 10.1158/1541-7786.MCR-23-0015 37327051

[40] Zeng K Xie W Wang C Wang S Liu W Su Y. USP22 upregulates ZEB1-mediated VEGFA transcription in hepatocellular carcinoma. Cell Death Dis (2023) 14(3):194. doi: 10.1038/s41419-023-05699-y 36906615 PMC10008583

[41] Hang X Zhao L Wu B Li S Liu P Xu J. BCL-2 isoform β promotes angiogenesis by TRiC-mediated upregulation of VEGF-A in lymphoma. Oncogene (2022) 41(28):3655–63. doi: 10.1038/s41388-022-02372-0 35701534

[42] Moraes L Piqueras L Bishop-Bailey D. Peroxisome proliferator-activated receptors and inflammation. Pharmacol Ther (2006) 110(3):371–85. doi: 10.1016/j.pharmthera.2005.08.007 16168490

[43] Wagner K Wagner N. Peroxisome proliferator-activated receptor beta/delta (PPARbeta/delta) acts as regulator of metabolism linked to multiple cellular functions. Pharmacol Ther (2010) 125(3):423–35. doi: 10.1016/j.pharmthera.2009.12.001 20026355

[44] Zhou Z Ma D Li P Wang P Liu P Wei D. Sirt1 gene confers Adriamycin resistance in DLBCL via activating the PCG-1α mitochondrial metabolic pathway. Aging (2020) 12(12):11364–85. doi: 10.18632/aging.103174 PMC7343448 32570218

[45] Gallorini M Di Valerio V Bruno I Carradori S Amoroso R Cataldi A. Phenylsulfonimide PPARα Antagonists enhance nrf2 activation and promote oxidative stress-induced apoptosis/pyroptosis in MCF7 breast cancer cells. Int J Mol Sci (2023) 24(2):1316. doi: 10.3390/ijms24021316 36674831 PMC9864319

[46] Li G Li X Mahmud I Ysaguirre J Fekry B Wang S. Interfering with lipid metabolism through targeting CES1 sensitizes hepatocellular carcinoma for chemotherapy. JCI Insight (2023) 8(2):e163624. doi: 10.1172/jci.insight.163624 36472914 PMC9977307

[47] Xu R Luo X Ye X Li H Liu H Du Q. SIRT1/PGC-1α/PPAR-γ Correlate with hypoxia-induced chemoresistance in non-small cell lung cancer. Front Oncol (2021) 11:682762. doi: 10.3389/fonc.2021.682762 34381712 PMC8351465

[48] Zhao Q Zhong J Lu P Feng X Han Y Ling C. DOCK4 is a platinum-chemosensitive and prognostic-related biomarker in ovarian cancer. PPAR Res (2021) 2021:6629842. doi: 10.1155/2021/6629842 33613670 PMC7878079

[49] Ishtiaq S Arshad M Khan J. PPARγ signaling in hepatocarcinogenesis: Mechanistic insights for cellular reprogramming and therapeutic implications. Pharmacol Ther (2022) 240:108298. doi: 10.1038/nm.3159 36243148

[50] Huang M Chen L Mao X Liu G Gao Y You X. ERRα inhibitor acts as a potential agonist of PPARγ to induce cell apoptosis and inhibit cell proliferation in endometrial cancer. Aging (2020) 12(22):23029–46. doi: 10.18632/aging.104049 PMC7746384 33197888

[51] Herpers B Eppink B James MI Cortina C Cañellas-Socias A Boj SF. Functional patient-derived organoid screenings identify MCLA-158 as a therapeutic EGFR × LGR5 bispecific antibody with efficacy in epithelial tumors. Nat Cancer (2022) 3(4):418–36. doi: 10.1038/s43018-022-00359-0 35469014

