# Supplementary material for: Modeling of senescence-related chemoresistance in ovarian cancer using data analysis and patient-derived organoids
Source: Front Oncol. 2024 Feb 2;13:1291559. doi: 10.3389/fonc.2023.1291559 (PMC10869451; doi:10.3389/fonc.2023.1291559)
Supplement: Supplementary file 1 [file DataSheet_1.docx]

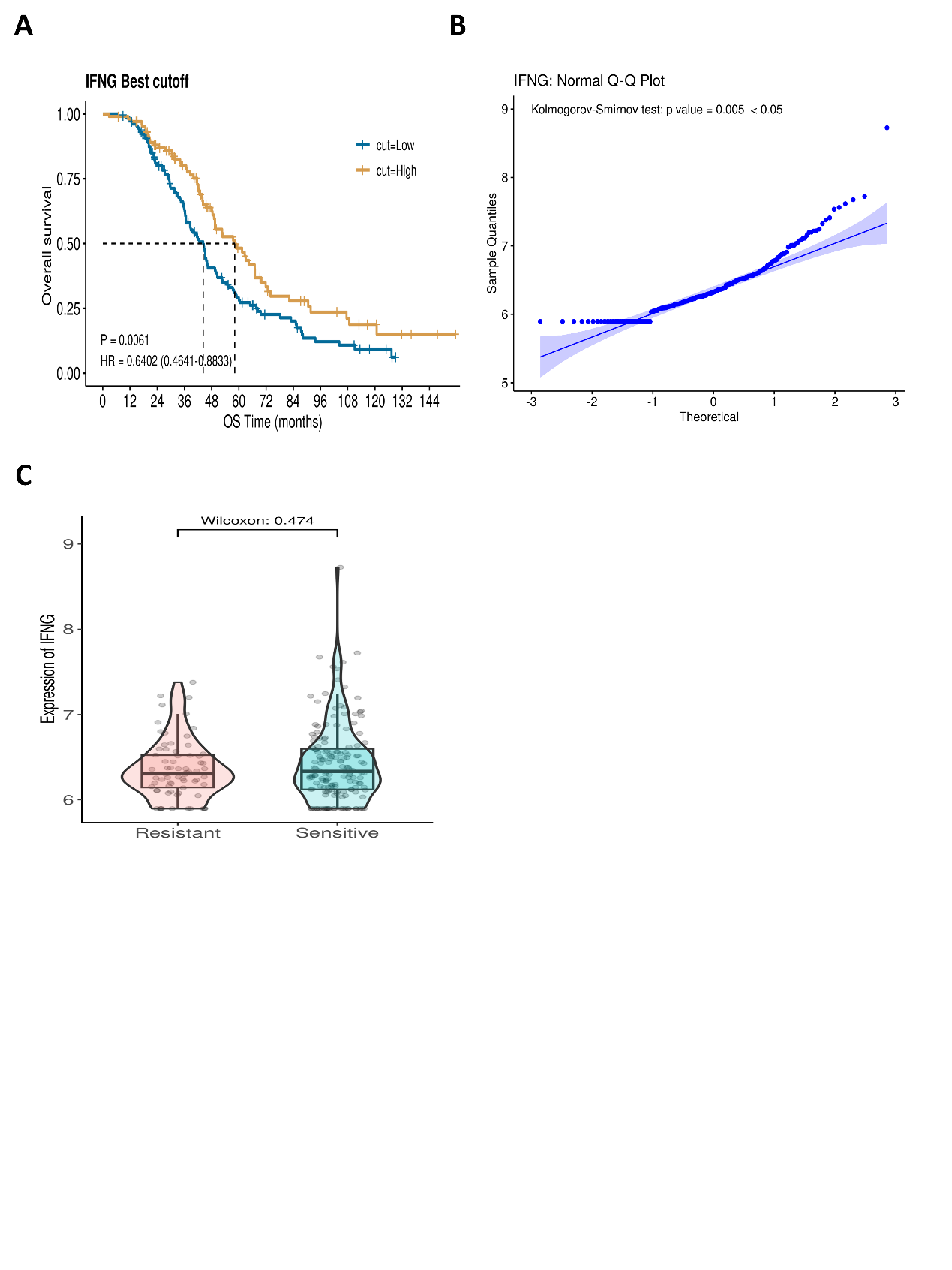


**Figure S1:** (A) The survival ability of OC patients with different high and low expression level of IFNG (*P*=0.0061). (B) Q-Q plot of IFNG to test the normal distribution of data. (C) The expression of IFNG in resistant and sensitive groups.

Supplementary Table 1. Clinical pathological parameters of OC patients.

| Clinical | Characteristics | n |
| --- | --- | --- |
| Survival Status | Alive | 115 |
|  | Dead | 172 |
| Platinum Status | Sensitive | 197 |
|  | Resistant | 90 |
| Overall Survival | <3y | 133 |
|  | ≥3y and <5y | 100 |
|  | ≥5y and <10y | 52 |
|  | ≥10y | 2 |
| Tumor stage | II | 14 |
|  | IIIA-IIIB | 13 |
|  | IIIC | 218 |
|  | IV | 42 |

Supplementary Table 2. Multiple Cox analysis results of model genes.

| Gene | Coef | HR | HR.95L | HR.95H | P |
| --- | --- | --- | --- | --- | --- |
| SGK1 | 0.26 | 1.3 | 1.1 | 1.6 | 0.0071 |
| VEGFA | -0.24 | 0.79 | 0.64 | 0.98 | 0.03 |
